# Hidden species diversity in an iconic living fossil vertebrate

**DOI:** 10.1098/rsbl.2022.0395

**Published:** 2022-11-30

**Authors:** Chase D. Brownstein, Daemin Kim, Oliver D. Orr, Gabriela M. Hogue, Bryn H. Tracy, M. Worth Pugh, Randal Singer, Chelsea Myles-McBurney, Jon Michael Mollish, Jeffrey W. Simmons, Solomon R. David, Gregory Watkins-Colwell, Eva A. Hoffman, Thomas J. Near

**Affiliations:** ^1^ Department of Ecology and Evolutionary Biology, Yale University, New Haven, CT 06520, USA; ^2^ Peabody Museum, Yale University, New Haven, CT 06520, USA; ^3^ North Carolina Museum of Natural Science, Raleigh, NC 27601, USA; ^4^ Department of Biological Science, The University of Alabama, Tuscaloosa, AL 35487, USA; ^5^ Museum of Zoology, University of Michigan, Ann Arbor, MI 48109, USA; ^6^ Florida Fish and Wildlife Research Institute, Milton, FL 32583, USA; ^7^ River and Reservoir Compliance Monitoring, Tennessee Valley Authority, Chattanooga, TN 37402, USA; ^8^ Department of Biological Sciences, Nicholls State University, Thibodaux, LA 70310, USA; ^9^ Division of Paleontology, American Museum of Natural History, New York, NY 10024, USA

**Keywords:** bowfin, ddRAD, phylogenetics, biogeography, species delimitation

## Abstract

Ancient, species-poor lineages persistently occur across the Tree of life. These lineages are likely to contain unrecognized species diversity masked by the low rates of morphological evolution that characterize living fossils. Halecomorphi is a lineage of ray-finned fishes that diverged from its closest relatives before 200 Ma and is represented by only one living species in eastern North America, the bowfin, *Amia calva* Linnaeus. Here, we use double digest restriction-site-associated DNA sequencing and morphology to illuminate recent speciation in bowfins. Our results support the delimitation of a second living species of *Amia*, with the timing of diversification dating to the Plio-Pleistocene. This delimitation expands the species diversity of an ancient lineage that is integral to studies of vertebrate genomics and development, yet is facing growing conservation threats driven by the caviar fishery.

## 1. Introduction

The bowfin, *Amia calva*, is the sole living representative of Halecomorphi, an ancient lineage of ray-finned fishes with a cosmopolitan distribution in the fossil record that is classically labelled as a living fossil clade [1–5]. Bowfin and the seven species of gars (Lepisosteidae) compose the ancient and species-depauperate Holostei, which is the sister lineage of the hyper-diverse Teleostei [4,6,7]. Together with sturgeons, the paddlefish, and mooneyes, bowfin and gars contribute to a hotspot of ancient vertebrate biodiversity in the species-rich temperate freshwater fish fauna of eastern North America [1,2,6,8–11].

Because of its evolutionary history, the bowfin is important for understanding genomic, developmental, and immunological evolution in vertebrates [1,4,7]. The bowfin is also notable for its apparently low rates of molecular evolution and phenotypic similarity to species from the fossil record dated to more than 145 Ma [1,2,4]. In addition, the economic significance of bowfin is increasing with an intensifying demand for sources of caviar [12], putting pressure on extant populations already strained by the centuries-long reputation of *Amia* as a ‘rough fish’ [13].

**Figure 1.**
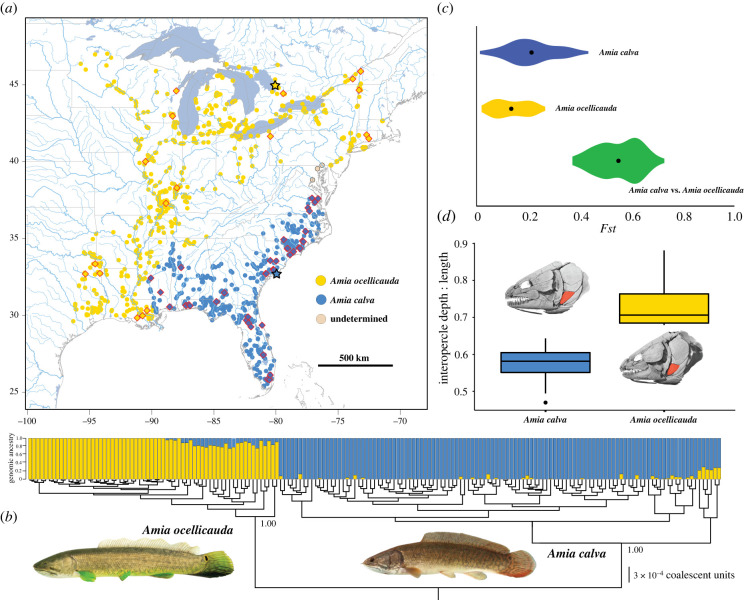
Identification of hidden bowfin species diversity. (*a*) Map of eastern North America showing museum specimen collection records of *Amia calva* (blue), *Amia ocellicauda* (yellow) and undetermined (tan), retrieved from fishnet2.net. Stars indicate type localities. Diamonds indicate specimens sampled in the ddRAD phylogenetic analysis. (*b*) Phylogeny and genomic structure analysis of 177 specimens of *Amia* based on 56 247 ddRAD loci. Photograph of *A. ocellicauda* from lower Tennessee River, Marshall County, Alabama, USA, YPM 035200, by J.M.M. and *Amia calva* from the Suwanee River, Gilchrist County, Florida, USA, UF 238466, by Z. Randall. (*c*) The comparison of pairwise *F*_st_ values for comparisons within *A. calva* (blue) and *A*. *ocellicauda* (yellow), and the comparisons between *A. calva* and *A*. *ocellicauda* (green). (*d*) Boxplot showing IO robusticity (ratio between maximum dorsoventral depth and maximum anteroposterior length) in *A. calva* and *A. ocellicauda*. CT-scanned skull of *A. calva,* TU 22613; CT-scanned skull of *A. ocellicauda,* TU 118772.

The bowfin has a wide geographical distribution across eastern North America (figure 1*a*) [2,14–16]. Among the species-rich fauna of North American freshwater fishes [17–19], relatively few species have geographical distributions as large as the bowfin. This wide geographical range includes many areas characterized by both high species diversity and a sizeable number of endemic freshwater fish species [20–23], enhancing the possibility of additional species diversity masquerading as *Amia calva* [24].

The taxonomic history of the bowfin provides another indication for the possibility of additional species in this clade. *Amia calva* was described by Linnaeus in 1766 [25] and in a period of 34 years between 1836 and 1870 [26–29], 12 species of *Amia* were described. At the close of the nineteenth century, all of these additional species were synonymized with *A. calva* without justification or reference to a study of variation among the named taxa [30, p. 113]. The only studies that explore genetic variation in *A. calva* sample a small part of the geographical distribution [24,31], and there is no study exploring variation in morphological traits among populations of *A. calva* across its entire geographical distribution [2,32] (figure 1*a*).

In this study, we analyse double-digest restriction-site-associated DNA (ddRAD) sequences from bowfin specimens sampled across their geographical distribution, using phylogenetic and population genetic techniques to look for the presence of distinct lineages (figure 1*b*). We also assess morphological variation in cranial features visualized with high-resolution computed tomography (CT) scans of *Amia* skulls and meristic trait data. The analysis of the genomic and phenotypic data is applied towards a delimitation of currently hidden species diversity in the sole living branch of the Halecomorphi.

## 2. Methods

### (a) Specimen sampling

Bowfin specimens were sampled over the course of several field seasons. Tissue samples were stored in 99% ethanol. Morphological voucher specimens were euthanized, fixed in an aqueous solution of formaldehyde for up to 21 days, soaked in tap water for up to 7 days, and transferred to 70% ethanol for long-term preservation in the ichthyology collection in the Yale Peabody Museum. Tissue samples were also obtained from museum collections. The sampling locations and museum collection records (if applicable) of all specimens used in the phylogenomic and morphological analyses are available on Dryad at http://doi.org/10.5061/dryad.8pk0p2nrf.

### (b) Generation of double-digest restriction-site-associated DNA loci

We extracted genomic DNA using a Qiagen DNeasy Tissue Extraction Kit (Qiagen, Valencia, CA, USA), following the manufacturer's protocols. The preparation of ddRAD libraries followed standard protocols [33], starting with approximately 400 ng of DNA from each specimen, and using *Pst*I/*Msp*I restriction enzymes. Size-selected libraries were sequenced using 100 bp single-end sequencing on an Illumina NovaSeq 6000 at the University of Oregon GC3F facility (http://gc3f.uoregon.edu). The demultiplexed reads were run through ipyrad v.0.9.68 [34], using default settings with the following exceptions: ‘reference’ for assembly method, ‘ddrad’ for datatype, ‘TGCAG, CCG’ for restriction overhang, ‘0.90’ for clustering threshold and ‘2’ for a stricter adapter filtration. The minimum number of specimens sharing a locus, hereafter referred to as ‘min’, was set to smallest numbers (184 and 75, see below) to reach a stationarity of loci dropout rate. ddRAD loci were aligned using the *A. calva* genome [4]. The raw sequence files for the ddRAD loci are available at Genbank (BioProject ID PRJNA868817).

### (c) Phylogenomic and population genomic analyses

The phylogenetic relationships among 177 sampled specimens of *Amia* were inferred from a concatenated DNA sequence dataset of the ddRAD loci. A posterior set of phylogenetic trees was generated using BEAST 2.6.4 [35] with a coalescent constant population size branching model, a GTR molecular evolutionary model with a gamma distribution of among-site rate variation, and a strict molecular clock model with a clock rate of 1.0. BEAST was run for 1.0 × 10^8^ generations and log and tree files were updated every 1.0 × 10^4^ generations. The convergence of parameter values was assessed by the effective sample sizes that were calculated using Tracer v.1.7 [36]. Generations sampled before convergence was attained were discarded as burn-in. The BEAST analyses were run three separate times and post-burn-in generations were pooled from all three runs using LogCombiner 2.8 [35]. A maximum clade credibility tree with median node heights was constructed for the post-burn-in species tree topologies using TreeAnnotator 2.6.4 [35].

Genomic differences at a subsample of 26 305 single nucleotide polymorphisms (SNPs) among populations were visualized in a principal components (PCs) analysis implemented in iPyrad v.0.9.68 [34]. The missing portion of the dataset was filled by a sample imputation method using the function ‘impute_method=sample’. Relative genomic ancestry was assessed using the ‘snmf’ function implemented in the R package LEA v.3.0.0 [37]. With the R package hierfstat [38], we estimated the fixation index (*F*_st_) for all pairs of specimens among the 177 sampled individuals.

### (d) Estimation of divergence times among living species of *Amia*

The divergence time of the two delimited species of *Amia* was estimated using a fossil tip dating strategy and the fossilized birth–death (FBD) branching model in BEAST 2.6.4 [35,39]. A total of 699 orthologous ddRAD loci were identified for the spotted gar (*Lepisosteus oculatus*) and the two delimited species of *Amia*. A single individual from each of the three sampled species was included in the FBD fossil tip dating analysis. The fossil lineages of Halecomorphi used in the tip dating analysis are listed in the electronic supplementary material, and their phylogenetic relationships were enforced with clade constraints that reflect relationships presented in phylogenetic analyses of living and extinct lineages in Holostei using morphological characters [2]. The prior settings for the FBD included an exponential distribution for the diversification rate and uniform distributions for the time of origin, sampling proportion and turnover parameters. The chain was run for 1.0 × 10^8^ generations and log and tree files were updated every 1.0 × 10^4^ generations. The convergence of parameter values was assessed by the effective sample sizes that were calculated using Tracer v.1.7. Generations sampled before convergence was attained were discarded as burn-in. The BEAST analyses were run three separate times and post-burn-in generations were pooled from all three runs using LogCombiner 2.8. A maximum clade credibility tree with median node heights was constructed for the post-burn-in species tree topologies using TreeAnnotator 2.6.4.

### (e) Assessment of disparity between delimited species of *Amia* in meristic traits

To investigate if disparity in meristic traits used to discover, delimit and describe species of fishes showed variation in *Amia*, we collected data from 225 specimens following standard protocols [2,40]. A PC analysis of the meristic traits was performed using the ‘prcomp’ function in R v.3.2.0 (http://www.R-project.org/). A cross-validation linear discriminant analysis (LDA) of the meristic data was conducted with the R package MASS (https://cran.r-project.org/web/packages/MASS/index.html).

### (f) Characterization of morphological differences in the skulls of delimited species of *Amia*

To further assess the presence of morphological differences between *A. calva* and delimited *A*. *ocellicauda*, we scanned eight specimens of *A. calva* and 12 specimens of *A*. *ocellicauda* using high-resolution CT with a Nikon XT H 225 ST system. All scan parameters are provided in the electronic supplementary material, table S1. Volume rendering was performed in VGStudio MAX 3.5.1 (volumegraphics.com). We used ImageJ to take digital measurements of CT scans digitally rendered in VGStudio MAX 3.5. Measurements were taken of the maximum depth and length of the subopercle and interopercle (IO), as well as of the number of alveoli in the dentary tooth row. All plots were made using ggplot2 in RStudio.

## 3. Results and discussion

The summarized posterior phylogeny resulting from the coalescent analysis of 56 247 ddRAD loci unambiguously resolves two major lineages in *Amia* (figure 1*b*): a clade that includes specimens from the type locality of *A. calva* Linnaeus [25, p. 500] in Charleston, South Carolina, USA and another that corresponds to a lineage for which the oldest available name is *Amia ocellicauda* Todd, in Richardson [29, p. 236]. The delimitation of the two species of *Amia* shows a break in the geographical distribution along the northern Gulf of Mexico (figure 1*a,b*). *Amia calva* is distributed from the Pearl River in Louisiana and Mississippi, USA to the Florida Peninsula, and the rivers draining to the Atlantic Ocean in Georgia, South Carolina, North Carolina and Virginia, USA (figure 1*a*). *Amia ocellicauda* was first described in 1836 from Lake Huron in Ontario, Canada [29] and is distributed from the Lake Pontchartrain system west in Gulf of Mexico draining rivers to the Colorado River system in Texas, USA, throughout the Mississippi River Basin, the Great Lakes Basin, the St Lawrence River system, including Lake Champlain, and the Atlantic draining Connecticut River system (figure 1*a,b*).

Patterns of genomic ancestry estimated in the snmf analysis demonstrate the genetic distinctiveness of the two species (figure 1*b*). Populations with signatures of admixture are those from the Gulf Coast that are reconstructed as early-branching in the coalescent model-inferred phylogenomic tree (figure 1*a,b*), which may result from incomplete lineage sorting or limited introgression following secondary contact. Most of the genetic variation in *Amia* was observed between the two species *A. calva* and *A. ocellicauda* (electronic supplementary material, figure S1). The mean pairwise *F*_st_ among all intraspecific comparisons of *A. calva* and *A*. *ocellicauda* were less than 0.25 and the average *F*_st_ value among all comparisons of *A*. *calva* and *A*. *ocellicauda* was greater than 0.55 (figure 1*c*), which is indicative of comparisons between species.

The geographical distribution of the two living species of *Amia* is suggestive of allopatric speciation associated with rivers draining into the Gulf of Mexico (figure 1*a*). The Bayesian FBD relaxed molecular clock analysis results in a mean posterior age estimate of 1.82 Ma, with a 95% credible interval ranging between 0.95 and 2.93 Ma, for the most recent common ancestor of the two species of *Amia*, suggesting the two species of *Amia* diverged during the Plio-Pleistocene. This timing of speciation is consistent with a pattern of glaciation-induced speciation along the Gulf Coast in other North American freshwater vertebrates [41–43].

The two delimited species are morphologically distinguished by a shape difference in the IO bone (figure 1*d*) and a diagnostic difference in the number of dentary teeth; *A. ocellicauda* has 15 teeth versus 16 or 17 teeth in *A. calva* (electronic supplementary material, table S2). Consistent with the observed lack of disparity in meristic traits (electronic supplementary material, tables S3 and S4), a PC analysis of the meristic traits shows substantial overlap of the two species when plotting PC2 versus PC1 (electronic supplementary material, figure S2). A cross-validation LDA of the meristic data shows that 88.9% of all *A. calva* specimens are correctly identified. By contrast, only 32.2% of the specimens of *A. ocellicauda* are correctly identified using the meristic trait data.

Our study reveals the presence of two recently diverged sibling species of bowfins. A recent study using ddRAD data, but with very limited sampling of *A. calva* and *A*. *ocellicauda*, concluded that there may be up to four living species of *Amia* [24]. With a more inclusive sampling of populations (figure 1*a*), our genomic analyses consistently delimit two species of *Amia*. The populations within either species that exhibit the greatest genetic divergence are those of *A*. *calva* from Florida and the Gulf Coast, which were not sampled in the other study [24]. In addition, the shape of the IO and the number of dentary teeth delimit two living species of *Amia* (figure 1*d*; electronic supplementary material, table S2).

Despite the extremely old age of their parent clade, *A. calva* and *A. ocellicauda* have diverged in the last two million years, contrasting with the view of the bowfin as an evolutionary ‘dead-end’ and recalling other ancient lineages that have more recently produced their standing species diversity [44,45]. A more accurate understanding of species diversity of bowfins will inform conservation decisions for this iconic living fossil lineage, which is the target of an emerging caviar fishery [12]. In turn, the illumination of hidden living diversity in bowfins demonstrates that North America has acted as both a cradle and refugium of ancient vertebrate diversity [2,4,6,9–11,46]. Along with evidence for deep splits in lineages of classic living fossils like coelacanths [47,48], the resurrection of *A. ocellicauda* reveals the potential for hidden species richness awaiting discovery in other deeply divergent and species-depauperate vertebrate lineages.

### (a) Taxonomy

***Amia calva*** Linnaeus 1766 [25, p. 500]

Ruddy bowfin, proposed common name

*Amia lintiginosa* Valenciennes in Cuvier & Valenciennes 1847 [26, p. 426].

*Amia cinerea* Valenciennes in Cuvier & Valenciennes 1847 [26, p. 430].

**Type material**: Linnean Society of London LSL128, a dried skin taken from the left side of the specimen [2, fig. 6].

**Diagnosis**: A species of *Amia* as previously diagnosed [2, pp. 32–33]. *Amia calva* has 16 or 17 teeth on the left side of the primary dentary tooth row (electronic supplementary material, table S2). IO maximum anteroposterior width measures 60% or less of IO dorsoventral depth. Colour ranges from reddish brown, and nuptial males may exhibit slightly green fins. Caudal eyespot is weakly or moderately defined. Meristic traits are summarized and compared with *A. ocellicauda* in electronic supplementary material, tables S3 and S4.

***Amia ocellicauda*** Todd in Richardson 1836 [29, p. 236]

Eyetail bowfin, proposed common name

*Amia occidentalis* De Kay 1842 [27, p. 269].

*Amia marmorata* Valenciennes in Cuvier & Valenciennes 1847 [26, p. 412].

*Amia ornata* Valenciennes in Cuvier & Valenciennes 1847 [26, p. 420].

*Amia viridis* Valenciennes in Cuvier & Valenciennes 1847 [26, p. 421].

*Amia canina* Valenciennes in Cuvier & Valenciennes 1847 [26, p. 424].

*Amia subcoerulea* Valenciennes in Cuvier & Valenciennes 1847 [26, p. 427].

*Amia reticulata* Valenciennes in Cuvier & Valenciennes 1847 [26, p. 431].

*Amia thompsonii* Duméril 1870 [28, p. 423].

*Amia piquotii* Duméril 1870 [28, p. 432].

**Type material**: There is no type specimen. In accordance with recommendations presented in Article 75 of the International Zoological Code of Nomenclature [49], we decline to designate a neotype specimen because there is no confusion regarding the taxonomic status of *A. ocellicauda*.

**Diagnosis**: A species of *Amia* as previously diagnosed [2, pp. 32–33]. *Amia ocellicauda* has 15 teeth on the left side of the dentary (electronic supplementary material, table S2). IO maximum anteroposterior width measures more than 60% of IO dorsoventral depth. Fin colour of nuptial males ranges from dull green to bright emerald green. Caudal eyespot is sharply defined in males. Meristic traits are summarized and compared with *A. calva* in electronic supplementary material, tables S3 and S4.

## Data Availability

All ddRAD, meristic and measurement data are either in the electronic supplementary material or are available from the Dryad Digital Repository: http://doi.org/10.5061/dryad.8pk0p2nrf [50]. Comprehensive methods are in the manuscript and electronic supplementary material, text [51].
